# Histone Maps in *Gossypium darwinii* Reveal Epigenetic Regulation Drives Subgenome Divergence and Cotton Domestication

**DOI:** 10.3390/ijms241310607

**Published:** 2023-06-25

**Authors:** Jinlei Han, Guangrun Yu, Xin Zhang, Yan Dai, Hui Zhang, Baohong Zhang, Kai Wang

**Affiliations:** 1 School of Life Sciences, Nantong University, Nantong 226019, China; 2 Department of Biology, East Carolina University, Greenville, NC 27858, USA

**Keywords:** histone modification, chromatin state, allotetraploid, domestication, *Gossypium darwinii*

## Abstract

The functional annotation of genomes, including chromatin modifications, is essential to understand the intricate architecture of chromatin and the consequential gene regulation. However, such an annotation remains limited for cotton genomes. Here, we conducted chromatin profiling in a wild allotetraploid cotton *Gossypium darwinii* (AD genome) by integrating the data of histone modification, transcriptome, and chromatin accessibility. We revealed that the A subgenome showed a higher level of active histone marks and lower level of repressive histone marks than the D subgenome, which was consistent with the expression bias between the two subgenomes. We show that the bias in transcription and histone modification between the A and D subgenomes may be caused by genes unique to the subgenome but not by homoeologous genes. Moreover, we integrate histone marks and open chromatin to define six chromatin states (S1–S6) across the cotton genome, which index different genomic elements including genes, promoters, and transposons, implying distinct biological functions. In comparison to the domesticated cotton species, we observed that 23.2% of genes in the genome exhibit a transition from one chromatin state to another at their promoter. Strikingly, the S2 (devoid of epigenetic marks) to S3 (enriched for the mark of open chromatin) was the largest transition group. These transitions occurred simultaneously with changes in gene expression, which were significantly associated with several domesticated traits in cotton. Collectively, our study provides a useful epigenetic resource for research on allopolyploid plants. The domestication–induced chromatin dynamics and associated genes identified here will aid epigenetic engineering, improving polyploid crops.

## 1. Introduction

In the eukaryotic nucleus, DNA is packaged into nucleosomes, which consist of histones that are subject to different types of post–translational modifications [1,2,3]. Histone modifications, including acetylation and methylation, are major regulators of chromatin structure and have various effects on gene transcription. In general, histone acetylation has an activating effect on transcription, whereas histone methylation can either be activating or repressive depending on the site and extent of the methylation [4,5]. For example, the acetylation of histone H3 at lysines 9 and 27 (H3K9ac, H3K27ac) is associated with active transcription. Similarly, the trimethylation of histone 3 at lysine 4 and 36 (H3K4me3, H3K36me3) leads to the activation of transcription, whereas the trimethylation of histone 3 at lysine 27 (H3K27me3) is associated with gene repression. Growing evidence has indicated that these histone modifications play an essential role in the growth, development, and adaptation to environmental fluctuation of the organism [6,7,8]. Aberrant histone modifications can cause severe developmental defects and have been linked to many diseases processes [9,10,11]. Therefore, they are indispensable for the normal survival of an organism.

Histone modification marks can act alone and collectively (known as the “histone code”) to co–regulate important biological processes [12,13,14]. In contrast to examining individual histone modifications, examining combinatorial and spatial patterns of multiple marks can provide additional information. Such patterns, termed “chromatin states”, often correspond to diverse classes of genomic elements, including promoters, enhancers, and transcribed, repressed, and repetitive regions [15,16]. So far, recognizing chromatin states along the genome has provided a systematic annotation of DNA elements in several model organisms, including humans [17,18], mice [19,20], Arabidopsis [21,22], rice [23], rapeseed [13], and wheat [14], which can then be integrated with gene expression data to generate new biological understanding. For example, a recent report on mouse liver regeneration defined six different chromatin states and revealed that pro–regenerative genes are maintained in active chromatin states but are restrained by H3K27me3, allowing for a rapid and synchronized response during regeneration [20]. In rice, phosphorus (P) starvation induced dramatic chromatin state transition from one state to another, which was correlated with the differential expression of P–deficiency–responsive genes [24].

Polyploidy is widespread in the plant kingdom, and polyploidization is regarded as the main force driving plant genome evolution and speciation [25,26]. Cotton (*Gossypium* spp.) is one of the most economically important crops in the world for the production of natural textile fibers and oilseed. There are approximately 45 diploid species (2n = 2x = 26) and 7 allotetraploid species (2n = 4x = 52) [27,28]. Two diploid species, *Gossypium arboreum* (Ga) and *Gossypium raimondii* (Gr), diverged from a common ancestor approximately 4.7–5.2 million years ago (Mya), and their genomes subsequently remerged via a polyploidization event approximately 1–1.6 Mya [29,30]. Thereafter, diversification and domestication occurred, finally leading to the development of seven distinct allotetraploids, including the wild *Gossypium darwinii* (Gd) and the cultivated *Gossypium hirsutum* (Gh). Thus, cotton is an excellent model plant species for obtaining genetic and epigenetic insights into polyploidization and domestication. In tetraploid cotton, biased gene expression between two subgenomes has been observed in diverse tissues [31], contributing to adaptation to diverse environments. Although histone modifications have been linked to the regulation of gene expression, the role of histone modifications in gene expression bias between two subgenomes in cotton is still largely unclear. Furthermore, domestication is accompanied by widespread changes in gene expression, and we still know very little about the significance of epigenetic mechanisms, specifically histone modifications, in regulating domestication.

Here, we generated chromatin immunoprecipitation sequencing (ChIP–seq) maps for five histone marks in Gd, including H3K4me3, H3K27me3, H3K36me3, H3K27ac, and H3K9ac. We discussed their intrinsic correlation with biased subgenome gene expression. Additionally, we defined six chromatin states by combining the profiles of histone modifications and open chromatin (DNase–seq) and investigated the impact of domestication on the chromatin states. Our datasets can be a valuable resource for functional genomics research on cotton, thereby benefiting its agronomic trait improvements.

## 2. Results

### 2.1. Genome–Wide Distribution of H3K4me3, H3K27me3, H3K36me3, H3K27ac, and H3K9ac in the Cotton Genome

**Figure 1:**
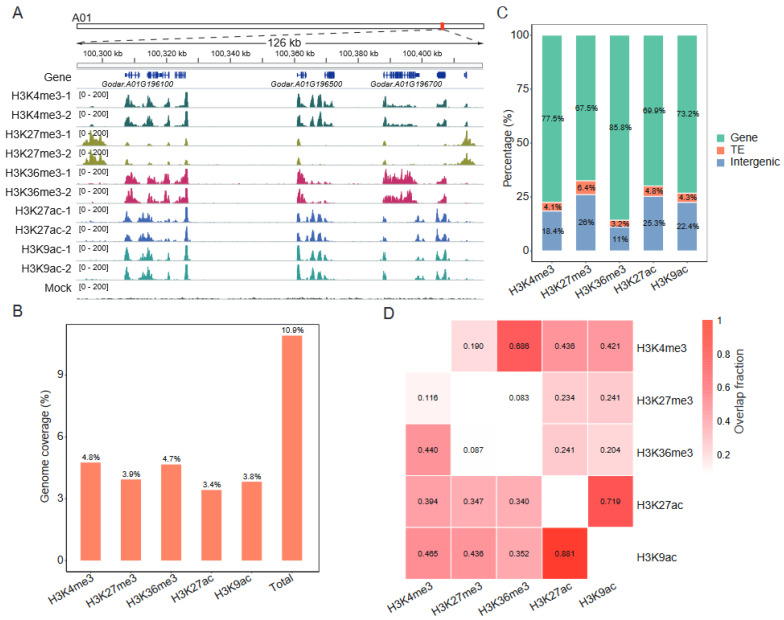
Genome–wide profiles of multiple histone modifications in the cotton genome: (**A**) Genome browser screenshot showing ChIP–seq data for H3K4me3, H3K27me3, H3K36me3, H3K27ac, and H3K9ac. Mock DNA was used as a control. (**B**) Genome coverage for H3K4me3, H3K27me3, H3K36me3, H3K27ac, and H3K9ac peaks. “Total” indicates the proportion of genome space with at least one histone modification. (**C**) The proportion of histone peaks categorized as genic, TE, or intergenic. (**D**) Relative proportion of histone peaks overlapped by other histone peaks. Peaks were considered overlapping if their coordinates overlapped by at least 50% of bases.

To investigate the biological role and significance of histone modifications in allotetraploid cotton, we conducted ChIP–seq using antibodies against H3K4me3, H3K27me3, H3K36me3, H3K27ac, and H3K9ac in young leaves of Gd. Two biological replicates were sequenced for each mark, and a total of 322.4 million paired–end reads were obtained (Supplementary Table S1). After mapping to the reference genomes, we obtained 225.7 million unique reads for downstream analyses. The biological replicates of each mark were well correlated (Pearson correlation coefficient ≥ 0.95) (Figure 1A, Supplementary Figure S1). Moreover, consistent with previous studies in other species [32], we found that the active histone marks H3K4me3, H3K27ac, H3K9ac, and H3K36me3 were enriched in the transcription start site (TSS) of active genes (Supplementary Figure S2). In contrast, the repressive mark H3K27me3 was enriched in inactive genes across the gene body. These results confirm the reliability of the data.

ChIP–seq peaks were then identified, and only consensus peaks that were detected in both two replicates were retained. In total, 26,388–47,764 peaks were identified (Supplementary Table S2). These peak regions covered 3.4–4.8% (from 74.7 Mb to 103.8 Mb) of the genome, and almost 11% (238.1 Mb) of the genome region was marked by at least one histone modification (Figure 1B). We further investigated the distribution of histone peaks on genome elements. It was found that more peaks lay within non–TE genes (67.5–85.8%) than on transposable elements (TEs) (3.2–6.4%) and intergenic regions (11.0–26.0%) for all marks (Figure 1C). Interestingly, H3K36me3 peaks showed a tight correlation with H3K4me3 (Figure 1D, Supplementary Figure S1), suggesting that they may be co–regulated and function together to control the transcriptional processes. In addition, a high co-localization between H3K27ac and H3K9ac was also observed. Compared with these four marks, the well–known repressive mark H3K27me3 was more highly enriched on TEs (Figure 1C), which is consistent with the fact that H3K27me3 participates in the regulation of TEs’ activity [33,34].

### 2.2. Distinct Combinatorial Patterns of Histone Modifications on Genes

**Figure 2:**
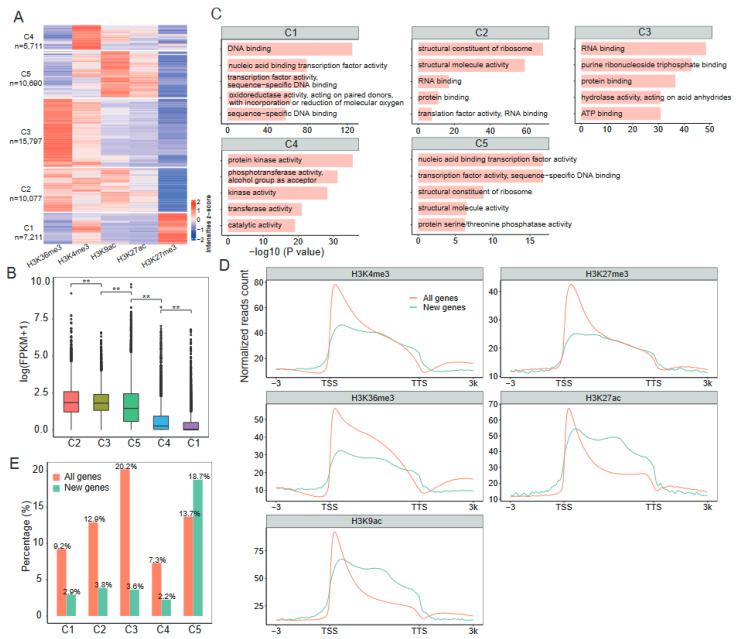
Clustering analysis reveals combinatorial association between histone modifications on genes: (**A**) Five clusters of genes marked by different combinations of histone modifications. The ChIP–seq signal intensities of each cluster of genes were calculated and normalized as RPKM for k–means clustering. The number of genes for each cluster is given. (**B**) Box plot presenting the distribution of gene expression levels for each cluster. The Wilcoxon test was used to analyze significance. “**” represents *p* value < 0.01. (**C**) Gene ontology (GO) analysis of genes in each cluster. The top 5 enriched GO molecular functions are indicated below each cluster. (**D**) Histone modification levels of the newly generated genes during cotton allopolyploidization. (**E**) The proportion of new genes within each of the five clusters. All genes in the genome are in the control group (Background).

To further characterize the patterns of histone modifications on genes, as well as their association with gene expression activities, we performed k–means clustering of genes with histone peaks within gene bodies, including 49,488 genes in total. This led to the identification of five clusters (C1–C5) of genes showing distinct histone modification states (Figure 2A, Supplementary Table S3). Cluster 1 (7211 genes) showed high levels of H3K27me3 and H3K4me3. Cluster 2 (10,077 genes) enriched H3K4me3, H3K36me3, and H3K9ac. Cluster 3 (15,797 genes) carried high levels of H3K36me3 and H3K4me3. Cluster 4 (5711 genes) was characterized by a prominent enrichment of H3K4me3 alone. Cluster 5 (10,690 genes) was preferentially marked by H3K9ac and H3K27ac. Overall, Cluster 2 genes showed significantly higher expression levels than those of genes in other clusters (*p* < 0.01, Wilcoxon test) (Figure 2B), similar to those reported in bread wheat [14]. In contrast, the low–expression genes were enriched in Cluster 1, consistent with the role of H3K27me3 in gene silencing. Furthermore, gene ontology (GO) enrichment analysis revealed that genes involved in transcription factor activity and oxidoreductase activity were enriched in Cluster 1. In contrast, genes involved in structural molecule activity and translation factor activity were enriched in Cluster 2 (Figure 2C). Cluster 3 genes were enriched by hydrolase activity. Cluster 4 genes were found to be enriched by protein kinase activity and transferase activity. Cluster 5 genes were enriched by phosphatase activity. Collectively, this analysis revealed that the combinatorial patterns of histone modifications constitute an epigenetic code that shapes gene expression states in specific functional categories.

### 2.3. Epigenetic Regulation of Newly Generated Genes during Cotton Allopolyploidization

The polyploidization of cotton resulted in the generation of new genes [35,36]. We wondered about the epigenetic features of these new genes. By BLASTP searches, we identified 4855 new genes in Gd with little or no similarities to the sequence of diploid progenitors *G. arboreum* and *G. raimondii* (coverage < 50% or identity < 50%). GO analysis revealed that these genes are mainly involved in photosynthesis and response to stress and stimulus (Supplementary Figure S3), which may explain the physiological changes between allotetraploid and diploid cotton, such as stress tolerance [37]. We then investigated the histone modification patterns around these new genes (Figure 2D). We found an overall increase in signal enrichment at new genes for each of the five histone marks we examined. However, when compared to the background of all genes, the average H3K4me3 signals were found to be lower in new genes. A similar pattern was also observed for the distribution of H3K27me3 and H3K36me3 (Figure 2D). In contrast, signals for H3K27ac and H3K9ac in new genes were higher than the background of all genes, except in the TSS regions (Figure 2D). This suggests that H3K27ac and H3K9ac may have a distinctive impact on the new genes.

Since cooperation between histone marks has been observed (Figure 2A), we next asked whether the new genes were associated with a particular combination pattern of histone modifications. We used all genes in the genome as a control. We found that 18.7% of new genes were grouped into Cluster 5 (Figure 2E), which was characterized by the simultaneous enrichment of H3K9ac and H3K27ac. This percentage was significantly higher than that of all genes (13.7%; *p* < 0.01, Fisher’s exact test). In contrast, a total of 12.6% of new genes were grouped into Clusters 1–4, whereas 49.5% of all genes were grouped into these four clusters (Figure 2E). Together, these findings suggest that new genes were more frequently embedded within a chromatin state with H3K9ac and H3K27ac co–modification.

### 2.4. Association between Subgenome Bias in Transcription and Histone Modifications

**Figure 3:**
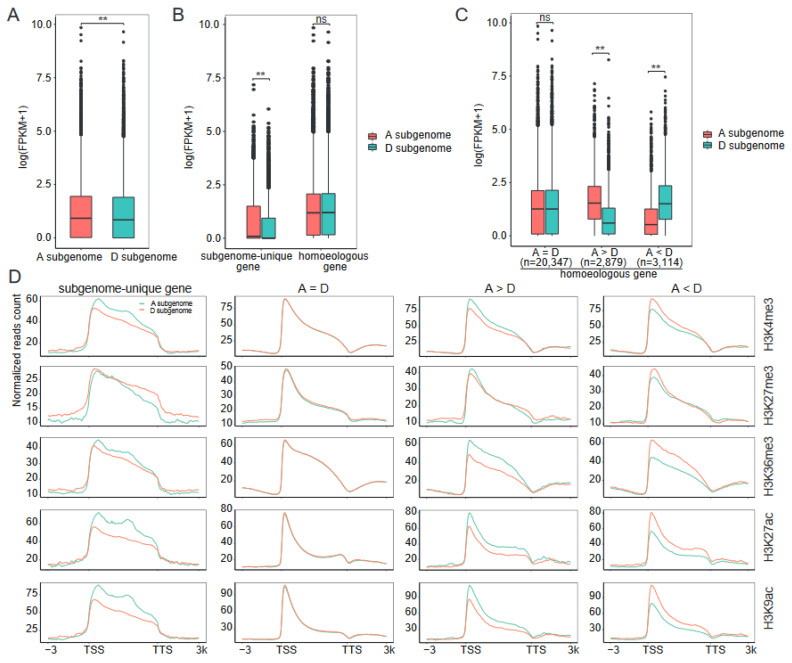
Histone modifications and gene expression differences between the A and D subgenomes: (**A**) Expression levels of genes in the A and D subgenomes. (**B**) Expression levels of homoeologous gene pairs and subgenome–unique genes in the A and D subgenomes. (**C**) Expression difference between homoeologous gene pairs. The homoeologous gene pairs were divided into three categories. A = D, A > D, and A < D indicate that homoeologous genes within the A subgenome show equal, higher, and lower expression levels than those within the D subgenome, respectively. The number of genes for each category is given. The Wilcoxon test was used to analyze statistical significance in (**A**–**C**). “**” represents *p* value < 0.01. “ns” represents no significance. (**D**) Histone modification levels of the A and D subgenomes in the genic regions.

We compared the average expression levels of genes between the two subgenomes of Gd, and found that they were higher in the A subgenome than the D subgenome genes (*p* < 0.01, Wilcoxon test) (Figure 3A). In addition, we observed that the average levels of the active marks H3K4me3, H3K36me3, H3K27ac, and H3K9ac on A subgenome genes were higher than those on D subgenome genes, whereas the average levels of the repressive mark H3K27me3 on A subgenome genes were lower than those on D subgenome genes (Supplementary Figure S4), suggesting a significant role of these marks in transcriptional bias to the A subgenome.

To further untangle the relationships between subgenome bias expression and histone modifications, we focused on homoeologous gene pairs and subgenome–unique genes between the two subgenomes. Using BLASTP searches (see Methods), 26,340 homoeologous gene pairs and 5849 subgenome–unique genes (2878 A–subgenome–unique genes and 2971 D–subgenome–unique genes) were obtained. GO analysis revealed that the homoeologous gene pairs from the A and D subgenomes tend to have similar functions, whereas the A– and D–subgenome–unique genes were enriched in different biological processes (Supplementary Figure S5). We found that the transcription bias of genes in the two subgenomes could primarily be attributed to the differential expression of the subgenome–unique genes (*p* < 0.01, Wilcoxon test) (Figure 3B). There were no significant differences in the average expression levels of the homoeologous gene pairs in the A and D subgenomes. However, by analyzing individual gene pairs, we found that 22.8% (5993/26,340) of the gene pairs showed expression bias (fold change ≥ 2 and false discovery rate (FDR) ≤ 0.05), including 2879 gene pairs with higher expression levels in the A subgenome than in the D subgenome (A > D) and 3114 gene pairs with higher expression levels in the D subgenome than in the A subgenome (A < D) (Figure 3C, Supplementary Table S4). An analysis of the histone modification levels at these genes revealed that similar gene expression levels correspond to similar levels of histone modification signals in the two subgenomes, and the genes with high expression levels showed high levels of H3K4me3, H3K36me3, H3K27ac, and H3K9ac (Figure 3D). Interestingly, we found that the average level of H3K27me3 on A–subgenome–unique genes was lower than that on D–subgenome–unique genes (Figure 3D). Consistent with this, the average expression level of A–subgenome–unique genes was higher than D–subgenome–unique genes (Figure 3B), whereas in homoeologous gene pairs, especially for A < D gene pairs, genes with high expression levels tend to have high levels of H3K27me3 (Figure 3D), suggesting that H3K27me3 plays different roles in the transcription of homoeologous gene pairs and subgenome–unique genes. Together, our findings demonstrate that the A subgenome exhibits higher levels of active histone marks, lower levels of the repressive histone mark, and higher gene expression compared to the D subgenome.

### 2.5. Defining Chromatin States in the Cotton Genome

**Figure 4:**
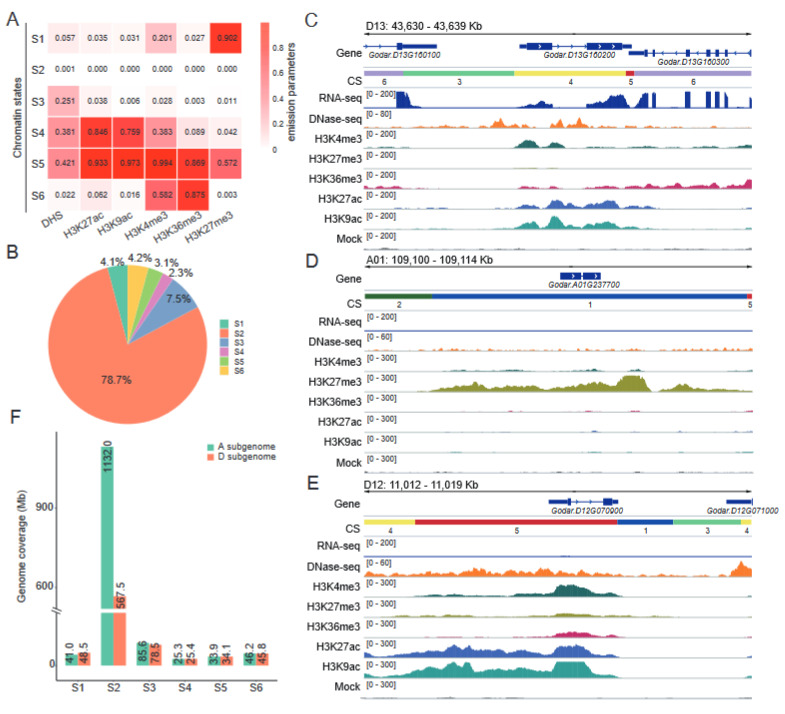
Chromatin state definitions and compositions in the cotton genome: (**A**) The heatmap presents the emission parameters for each chromatin state (S1–S6). Each row corresponds to one state, and each column corresponds to one chromatin marker. The emission parameters were determined with ChromHMM and represent the enriched possibility, indicated by values in each box. (**B**) The fraction of the cotton genome covered by each state. (**C**–**E**) Representative genes showing the pattern of CS on *Godar.D13G160200* (S4), *Godar.A01G237700* (S1), and *Godar.D12G070900* (S5). (**F**) Comparison of the coverage of each state between the A and D subgenomes.

We integrated the five histones’ ChIP–seq and DNase–seq datasets [32] to define distinct chromatin states (CSs), which can be used to characterize the functional activities of genomic sequences [13]. Based on the ChromHMM algorithm [18], the genome was classified into six distinct states (S1–S6), each of which could be distinguished from the others by differential enrichment of one or more of the marks considered in this study (Figure 4A). S1 was dominated by repressive mark H3K27me3. Three states (S3, S4, and S5) were characterized by the marks of open chromatin, of which S4 and S5 were also highly enriched for multiple histone marks, including H3K27ac and H3K9ac. S6 was enriched in both H3K4me3 and H3K36me3, but was inaccessible based on the absence of DNase–seq signal. Notably, S2 was devoid of any of the epigenetic marks examined. This state covers 78.7% of the total genome, similar to results in mice [20], whereas states representing open chromatin (S3–S5) account for 13.0% of the genome and the inaccessible chromatin states (S1 and S6) account for 8.3% of the genome (Figure 4B).

S4 and S5 were enriched for DNase–seq, H3K27ac, and H3K9ac, suggesting that actively transcribed genes would be found in these states. Genome browser views in Figure 4C illustrate the following patterns: genes such as *Godar.D13G160200* are a cotton homolog of the *Arabidopsis* gene *At3g12700* (*NANA*), which encodes an aspartic protease and plays an important regulatory function in chloroplasts that influence photosynthetic carbon metabolism [38]. This gene was highly expressed in the leaf tissues that characterize S4. In contrast, S1 was dominated by H3K27me3, the repressive mark suppresses the *Godar.A01G237700* gene (Figure 4D). Meanwhile, H3K27me3 was also detected in S5, suggesting that there were open chromatin regions that were repressed by H3K27me3. *Godar.D12G070900* represents such a bivalent gene (Figure 4E). We then compared the genome coverage of distinct CSs between subgenomes (Figure 4F). The CSs were largely similar between the two subgenomes, with the exception of S2. The genomic coverage of S2 (unmarked states) in the A subgenome was almost twice higher than that in the D subgenome, which could be attributable to the genome size of the A subgenome (~1364.1 Mb) being nearly twice that of the D subgenome (~799.8 Mb). Notably, we found that genomic coverages of S1 (inaccessible chromatin states) were higher in the D subgenome than in the A subgenome, whereas genomic coverages of S3 (open chromatin states) were higher in the A subgenome than in the D subgenome. Accordingly, the A subgenome was more active than the D subgenome at the CS level, which may explain why the A subgenome has high overall transcriptional activity (Figure 3A).

### 2.6. Chromatin States Associate with Genomic Elements and TEs Landscape

**Figure 5:**
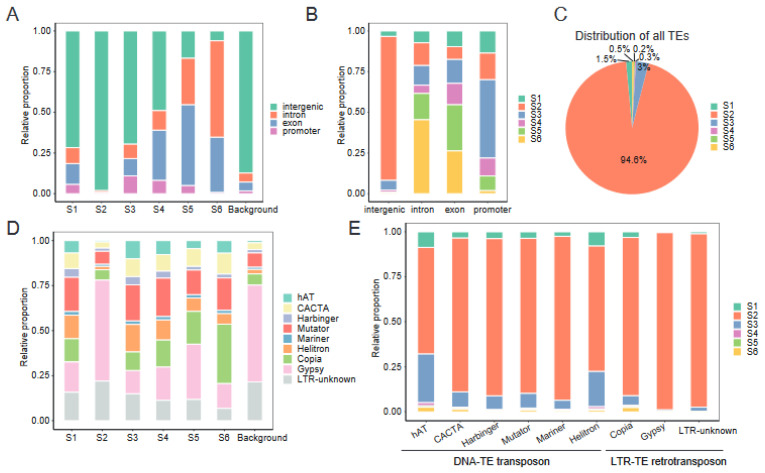
The distribution of chromatin states in diverse functional genomic elements: (**A**) Relative proportion of different genomic elements across each chromatin state. For comparison, “Background” represents the proportion of the genome that each element constitutes. (**B**) Relative proportion of different chromatin states across each genomic element. (**C**) Distribution of all TEs across each chromatin state. (**D**) Relative proportion of different TE classes across each chromatin state. For comparison, “Background” represents the proportion of the genome that each TE class constitutes. (**E**) Relative proportion of different chromatin states across each TE class.

If each CS serves distinct functional roles in the genome, the distribution of CSs across the genome should present several biases with genomic elements. To test this, we mapped the distribution of each CS across different genomic elements, including introns, exons, promoters (defined as −500 bp of transcription start site (TSS) of annotated genes), and intergenic regions. We observed that S3, S4, and S5 were enriched in promoter regions compared to the whole–genome background (Figure 5A), with over two thirds (68.2%) of all promoter regions in the genome occupied by S3–S5 combined, whereas only a small fraction (7.9%) of intergenic regions was present in these open states (Figure 5B). In contrast, S6 occupies less than 2% of all promoter regions and was enriched in intron and exon regions (Figure 5A,B). S1, which was characterized by high level of H3K27me3, covers 13.6% of all promoter regions and was enriched in exon regions, but has a relatively lower occupancy (3.4%) of intergenic regions (Figure 5A,B).

Furthermore, given that TEs are abundant (>60%) in the cotton genome and that TEs are thought to be silenced by epigenetic mechanisms [29,39], we wondered what the distribution of CSs over distinct TE classes was. We observed that the majority of TEs (94.6%) resided in S2, and there were less than 6% of TEs in other CSs (Figure 5C). This was logical, since S2 encompasses the vast majority of the genome, especially for intergenic regions (Figure 4B and Figure 5B). Consistent with this, the distribution of distinct TE classes in S2 was essentially the same as the whole–genome background (Figure 5D). Gypsy–type TEs were depleted from S1, S3, and S6, although Gypsy is the dominating type of TE in the whole genome, but were enriched in S2 (Figure 5D,E). Less than 7% of all TEs are Copia, whereas these represented the largest category of TEs in S6. S1 was selectively enriched with DNA transposon, such as hAT and Helitron elements, which suggest that these TEs were sequestered in heterochromatin. Together, these biases in the distribution provide evidence for CSs’ involvement in cotton genome organization.

### 2.7. Domestication Has a Dramatic Impact on Chromatin States

**Figure 6:**
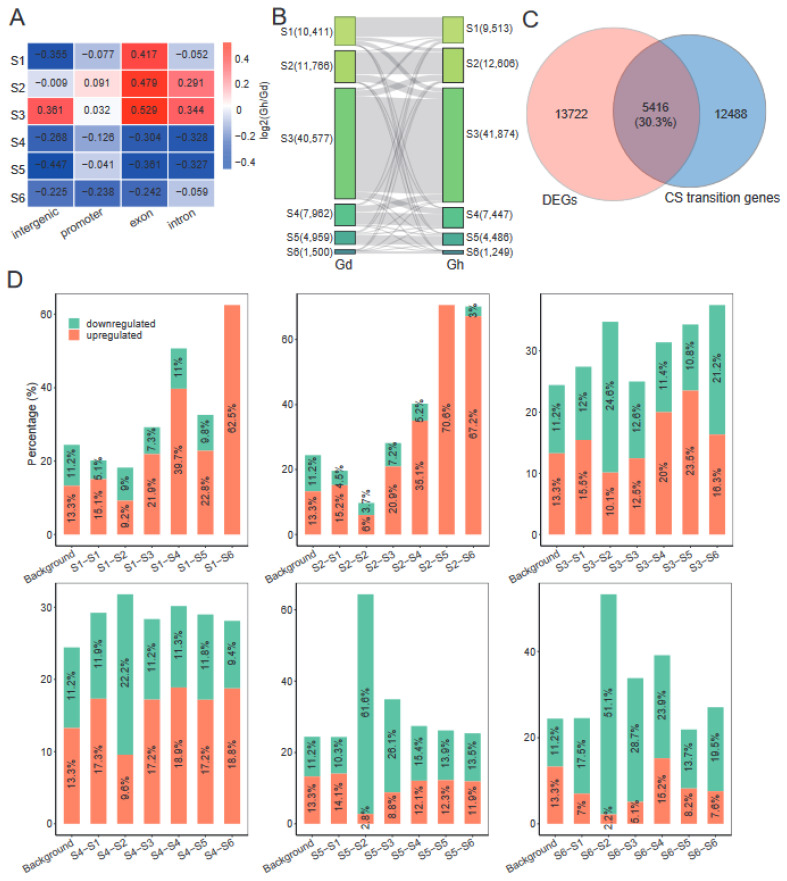
Dynamic changes in chromatin states and gene expression during cotton domestication: (**A**) Fold changes of CS coverage in each genomic element between the Gh and Gd samples. Red and blue colors show increased and decreased enrichment during domestication. (**B**) CS transitions of genes from Gd to Gh. The number of genes for each CS is given. The width of each ribbon represents the number of genes with a transition to another CS. (**C**) Venn diagram showing the overlap between CS transition genes and DEGs. (**D**) Bar plot showing the overlap between CS transition genes and upregulated or downregulated DEGs. The overall prevalence of upregulated or downregulated DEGs in the genome was used as a control (Background).

Next, we compared chromatin states between Gd and cultivated tetraploid cotton *G. hirsutum* (Gh) [32] to characterize the impact of domestication on CSs. To improve data comparability, we mapped each dataset to the reference sequences of Gd, as shown previously [32]. The CS models were similar between Gh and Gd (Supplementary Figure S6). We then compared the distribution of CSs between Gh and Gd within different genomic elements, and found that S4–S6 decreased and S3 increased in promoter, exon, intron, and intergenic regions (Figure 6A). In contrast, S1 decreased in promoter, intron, and intergenic regions, but increased in exons, whereas S2 increased in promoters, exons, and introns and decreased in intergenic regions. Because the promoter is an important regulatory region that controls gene expression, we investigated the effect of domestication at the promoter in more detail. We observed that 23.2% of genes in the genome exhibited a CS transition at their promoter during domestication (Figure 6B, Supplementary Table S5). Among them, the largest groups of transitions were S2–S3 (2617 genes), S4–S3 (2355 genes), S3–S2 (2330 genes), and S3–S4 (2160 genes). GO enrichment analysis revealed that different biological processes were enriched in different transition groups (Supplementary Figure S7). For example, S2–S3 genes were enriched for the light reactions of photosynthesis and the regulation of flower and shoot system development, whereas S4–S3 genes were enriched in several important metabolic processes, such as the ATP metabolic process. Notably, the terms related to the response to abiotic stimuli and photosynthesis, including light harvesting, light reaction, and photosystem II assembly, were found to be significantly enriched in S3–S4 genes. These CS transition genes could contribute to morphological or physiological changes, such as fiber traits and photoperiod sensitivity, during cotton domestication [40,41].

We investigated the relationship between gene expressions and CS transitions during domestication by comparing the RNA–seq data of Gh [32]. Differential expression analysis identified 19,138 differentially expressed genes (DEGs) during domestication (fold change ≥ 2 and false discovery rate (FDR) ≤ 0.05), including 10,395 upregulated and 8743 downregulated genes (Supplementary Table S6). We examined the overlap between CS transition genes and DEGs, and found that 30.3% of CS transition genes were DEGs (Figure 6C). This frequency was significantly higher than the overall prevalence of DEGs (24.4%, the percentage of DEGs relative to total genes) (*p* < 0.01, Fisher’s exact test), suggesting that differential expression during domestication correlated with CS transitions. Meanwhile, we quantified the overlap between the upregulated or downregulated DEGs and each transition group (Figure 6D). These analyses revealed some obvious biases between DEGs and CS transitions. For example, upregulated DEGs were enriched in S2–S3, S2–S4, S2–S5, and S2–S6 transitions (i.e., transitions from an epigenetic–mark–deficient state to an active–mark–enriched state), which is consistent with increased gene expression being correlated with a deposition of active epigenetic marks. Reciprocally, downregulated DEGs were enriched among S3–S2, S4–S2, S5–S2, and S6–S2 transitions. Thus, these results highlight that specific chromatin dynamics and epigenetic regulation may play important roles during cotton domestication.

## 3. Discussion

Histone modification is one of the most common types of epigenetic modification and has been shown to play an important role in the regulation of chromatin structure and gene transcription in mammals and plants. Examining histone modifications will help us comprehensively understand the functional and structural properties of the genome. Gd is a typical allopolyploid plant, which contains two divergent subgenomes and has a complex genome structure, and still lacks comprehensive epigenetic modification information. In this work, we present a comprehensive report of five histone modifications in the Gd using ChIP–seq, which can serve as a valuable resource for studying the epigenetic characteristics of allopolyploid plant genomes, the imbalance between subgenomes, and cotton domestication.

We annotated nearly 11% of the Gd genome based on ChIP–seq profiles (Figure 1B). Similar to the pattern in mammals and other plant species [14,32,42,43], H3K4me3, H3K36me3, H3K27ac, and H3K9ac correlate with transcription activation and tend to be restricted to the gene regions, especially in TSS (Figure 1C, Supplementary Figure S2). This suggests that their functions are mostly at the TSS of the gene. H3K27me3 is also abundant at the TSS, but appears to play roles in gene silencing by deposition across gene bodies (Supplementary Figure S2). Interestingly, our data show that H3K27me3 deposition around the TSS correlates with highly transcribed genes. Previous studies in mouse embryonic stem cells have reported a similar phenomenon [44], where H3K27me3 enrichment in the promoter is associated with active transcription, suggesting that these regions might serve as bivalent domains harboring both repression and activation marks (such as H3K4me3 and H3K36me3). Indeed, the clustering of genes based on histone modification levels confirms that several genes, for example Cluster 1 genes, were marked by H3K27me3, either alone or bivalently with H3K4me3 (Figure 2A), which may help to explain their transcriptional activity.

From the RNA–seq perspective, our data show that the A subgenome is the dominant one in Gd (Figure 3A). The most significant differences between the A and D subgenomes are in the subgenome–unique genes, and the average expression of homoeologous gene pairs from two subgenomes tends to be in a balanced pattern (Figure 3B). Subgenome–unique genes in the A subgenome exhibit significantly higher transcriptional levels than those in the D subgenome, which is consistent with higher levels of active marks and lower levels of repressive marks in the A subgenome (Figure 3D). It is noteworthy that a similar pattern was observed in *Brassica napus* [13], an allopolyploid species harboring An and Cn subgenomes. In *Brassica napus*, the expression levels of genes in the An subgenome were found to be significantly higher compared to those in the Cn subgenome. This asymmetrical transcriptional activity between the An and Cn subgenomes can be primarily attributed to the distinct expression patterns exhibited by the subgenome–unique genes, rather than the homoeologous gene pairs between the An and Cn subgenomes. Additionally, it has been demonstrated that epigenetic modifications play a crucial role in influencing this phenomenon. These intriguing findings shed light on the distinctive regulation of subgenome–unique genes and the remarkable balance in the expression of homoeologous gene pairs, emphasizing the prevalence of these phenomena across different allopolyploid species. On the other hand, a number of studies have identified and demonstrated the significant regulatory roles of CSs in eukaryotic gene expression [20,24,45]. We further combined histone marks data from this study with our previous open chromatin (DNase–seq) data [32] to define six CSs (S1–S6) in Gd (Figure 4), thereby extending the existence of epigenetic code to Gd. As in mouse [20], wheat [14], and rapeseed [13], CSs were efficient for predicting genomic elements and TEs landscape (Figure 5). Between–subgenome comparison analyses revealed that the coverage of S1 (defined by the enrichment of H3K27me3, inaccessible chromatin states) in the D subgenome was 48.5 Mb, which was higher than the 41.0 Mb in the A subgenome (Figure 4F). The higher H3K27me3 enrichment and lower chromatin accessibility in the D subgenome may be important factors contributing to the gene expression bias toward the A subgenome. This suggests that the imbalance between the A and D subgenomes might be attributed to the differences in the epigenetic states.

Our work has also shown the importance of epigenetic regulation in cotton domestication. We compared CS changes between cultivated cotton (*G. hirsutum*) and wild cotton (*G. darwinii*). More than 23% of genes in the genome exhibited a CS transition at their promoter during domestication (Figure 6B), and these transitions correlated with genes differentially expressed during domestication (Figure 6C). Many CS transition genes, for example in the S2–S3 and S3–S4 groups, were predicted to be involved in photosynthesis (Supplementary Figure S7), and thus might contribute to the changes in photoperiod sensitivity in cultivated cotton, a major domestication syndrome trait of cotton [40,46]. Future functional experiments on these genes would shed light on this hypothesis and ultimately improve our understanding of the role of epigenetics in the domestication of cotton and other polyploid crops. Additionally, it is noteworthy that, although we demonstrated that there were significant correlations between CS transition genes and DEGs statistically (*p* < 0.01, Fisher’s exact test), there were many genes exhibiting a CS transition that were not DEGs (Figure 6C). This likely reflects the presence of other molecular processes, such as the binding of appropriate transcription factors or other epigenetic modifications (such as DNA methylation), which determine steady–state transcription levels. Nevertheless, the correlations between changes in CSs and gene expression highlight the importance of epigenetic regulation in differential gene expression during cotton domestication.

Overall, we generated a comprehensive dataset regarding histone modification in Gd. This will be a valuable resource for the polyploid plant epigenetics research community, and will help identify desirable epigenetic elements to assist in crop improvement by epibreeding.

## 4. Materials and Methods

### 4.1. Plant Materials and Growth Conditions

Gd seeds were sterilized and grown under environment–controlled greenhouse conditions (13 h/11 h of light/dark, 28 °C light/26 °C dark, 60% humidity). The third and fourth true leaves were harvested (when the fifth true leaf emerged) and frozen immediately in liquid nitrogen for the ChIP–seq and RNA–seq experiments.

### 4.2. ChIP–Seq and Data Analysis

ChIP–seq assay was performed using about 2 g leaves as previously described [32]. Five commercial antibodies against H3K4me3 (Cat. no. 07–473, Millipore Sigma, St. Louis, MO, USA), H3K27me3 (Cat. no. 07–449, Millipore Sigma, St. Louis, MO, USA), H3K36me3 (Cat. no. ab9050, Abcam, Cambridge, MA, USA), H3K27ac (Cat. no. 07–360, Millipore Sigma, St. Louis, MO, USA), and H3K9ac (Cat. no. 06–942, Millipore Sigma, St. Louis, MO, USA) were used in immunoprecipitation. The Mock DNA was purified as a control. Illumina sequencing libraries were prepared from the ChIP and Mock DNAs by adaptor ligation, size selection, and PCR amplification. The resulting libraries were sequenced using an Illumina NovaSeq 6000 system (150 bp paired–end mode) (Illumina, San Diego, CA, USA). Two biological replicates were performed for each ChIP–seq experiment. The raw reads obtained from sequencing were quality–filtered and trimmed by using trim_galore v.0.6.7 package (accessed on 10 September 2022, https://www.bioinformatics.babraham.ac.uk/projects/trim_galore/). The remaining cleaned reads were mapped to the Gd reference genome using Bowtie2 v.2.2.5 [47] with default parameters. The genome sequence and annotation files for Gd (HGS–v1.1) were downloaded from CottonGen (accessed on 10 September 2022, https://www.cottongen.org/). Mapped reads were then filtered using SAMtools v.1.9 [48] with the parameters “-f 2 -F 2048 -q 10” to retain only correctly read pairs with a mapping quality score of 10 or higher for further analysis. Peak calling was performed by the MACS2 v.2.1.4 [49] with the parameters “-t ChIP.bam -c Mock.bam -f BAMPE --broad -g 2.1 × 10^9^ --nomodel” and using the following criteria: *p* value < 1× 10^−10^ and fold change > 5. Only consensus peaks detected in both replicates were retained for downstream analyses. Peaks were annotated to genomic regions (gene, TE, and intergenic region) according to the genomic position of their midpoint by intersect function in BEDTools v.2.26.0 [50].

### 4.3. RNA–Seq and Data Analysis

Total RNA from leaves was extracted using the Omega Plant RNA kit (Cat. no. R6827–01, Omega Bio-tek, Doraville, GA, USA) following the manufacturer’s instructions. RNA–seq libraries were constructed using the Illumina TruSeq RNA Kit (NEB, Cat. no. E7530) and were sequenced with an Illumina NovaSeq 6000 system (150 bp paired–end mode). Two biological replicates were performed for RNA–seq experiment. RNA–seq sequencing reads were cleaned as described above. The clean reads were then mapped to the reference genome using Tophat2 v.2.1.1 [51] with default settings. The Cufflinks v.2.2.1 [52] was employed to calculate the gene expression levels represented by FPKM (fragments per kilobase of transcript per million mapped reads) values. To compare expression levels across samples and genes, FeatureCounts v.2.0.1 [53] was used to calculate the read counts per gene and differential expression analysis was performed using DEseq2 [54] based on threshold criteria of an adjusted *p*–value  < 0.05 and |log2(fold change)|  >  1. Gene ontology (GO) enrichment analysis was performed by using an online resource (accessed on 24 December 2022, www.omicshare.com/tools) with default instructions.

### 4.4. TE Analysis 

TEs were identified in the Gd reference genomes by using the EDTA v.1.9.6 pipeline [55]. The annotation table from EDTA was then parsed and hierarchically classified into two major classes: retrotransposons (including Gypsy, Copia, etc.) and DNA transposons (including hAT, CACTA, etc.).

### 4.5. Definition of Homoeologous Gene Pairs and Subgenome–Unique Genes

Homoeologous gene pairs were identified as previously described [31]. Briefly, the protein sequences of the genes from the A and D subgenomes were used as queries in BLAST searches against one another. Gene pairs that displayed the best reciprocal BLAST hits (E–value < 1 × 10^−5^, coverage >= 50%, identity >= 50%) between the two subgenomes were identified as homoeologous. In contrast, genes with little or no sequence similarities between the two subgenomes (coverage < 50% or identity < 50%) were identified as subgenome–unique genes. Thus, 26,340 homoeologous gene pairs and 5849 subgenome–unique genes (2878 A–subgenome–unique genes and 2971 D–subgenome–unique genes) were obtained.

### 4.6. DNase–Seq Data Analysis

DNase–seq data of Gd leaf tissue were obtained from our previous publication (European Nucleotide Archive (ENA) accession number: PRJEB47222) [32]. The data processing, including read cleaning and mapping steps, was the same as described in our previous work [32]. Only correctly read pairs with a mapping quality score >= 10 were retained for further analysis.

### 4.7. Chromatin States Analysis

We used the program ChromHMM v.1.23 [18], which is based on a multivariate Hidden Markov Model, to characterize the CS maps for Gd. The filtered BAM files for the five histones’ ChIP–seq and DNase–seq data were binarized using ChromHMM BinarizeBam function with the parameters “-paired -b 200 -f 2”. Six CSs were generated based on the LearnModel function in ChromHMM. To characterize the impact of domestication on CSs and gene expression, ChIP–seq (H3K4me3, H3K27me3, H3K36me3, H3K27ac, and H3K9ac), DNase–seq, and RNA–seq data from leaf tissue of domesticated tetraploid cotton *G. hirsutum* (Gh) were obtained from our previous publication (ENA accession number: PRJEB47222) [32]. To improve data comparability, reads from Gh were mapped to the reference sequence of Gd. CSs were predicted as described above. Each gene was assigned to one CS based on the state of the gene’s promoter (defined as −500 bp of TSS). When multiple states were found for a single promoter, the longer CS was counted.

### 4.8. Data Visualization

For visualization, the filtered BAM files were converted to the bigwig format by using the bamCoverage function in deepTools v.3.1.3 [56] with a bin size of 10 bp and RPKM normalization. The metagene profiles of ChIP–seq data were generated using the computeMatrix and plotProfile functions in the deepTools package. Genome browser images were created by using the Integrative Genomics Viewer (IGV) v.2.3.92 software (accessed on 15 September 2022, https://software.broadinstitute.org/software/igv/) with bigwig files processed as described above.

## Data Availability

The ChIP–seq and RNA–seq data described in this work have been deposited to the Genome Sequence Archive (GSA) database (accessed on 10 January 2023, http://gsa.big.ac.cn/) under the accession number PRJCA015055.

## Supplementary Materials

The following supporting information can be downloaded at https://www.mdpi.com/article/10.3390/ijms241310607/s1.
