# Supplementary material for: Histone Maps in Gossypium darwinii Reveal Epigenetic Regulation Drives Subgenome Divergence and Cotton Domestication
Source: Int J Mol Sci. 2023 Jun 25;24(13):10607. doi: 10.3390/ijms241310607 (PMC10341804; doi:10.3390/ijms241310607)
Supplement: Supplementary file 1 [file ijms-24-10607-s001.zip › Supplementary Fig 1-7.pdf]

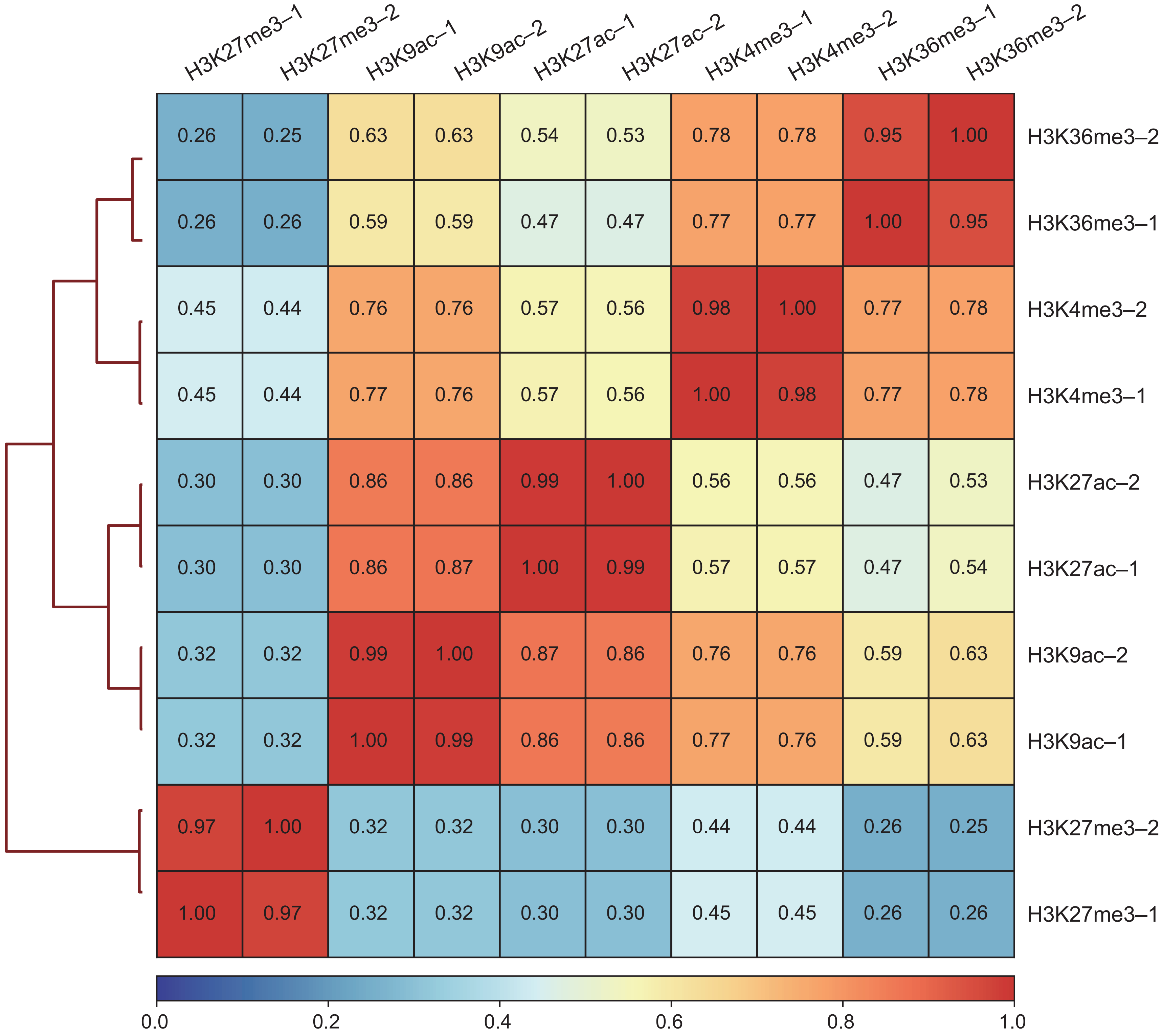

Supplementary Figure 1. Pearson correlations of ChIP–seq replicates.
The genome was divided into nonoverlapping 200–bp windows, and the numbers of ChIP–seq reads in each window were calculated. The correlation heatmap was generated with deepTools software. The colors represent the correlation coefficients, with red indicating high similarity and blue indicating low similarity.

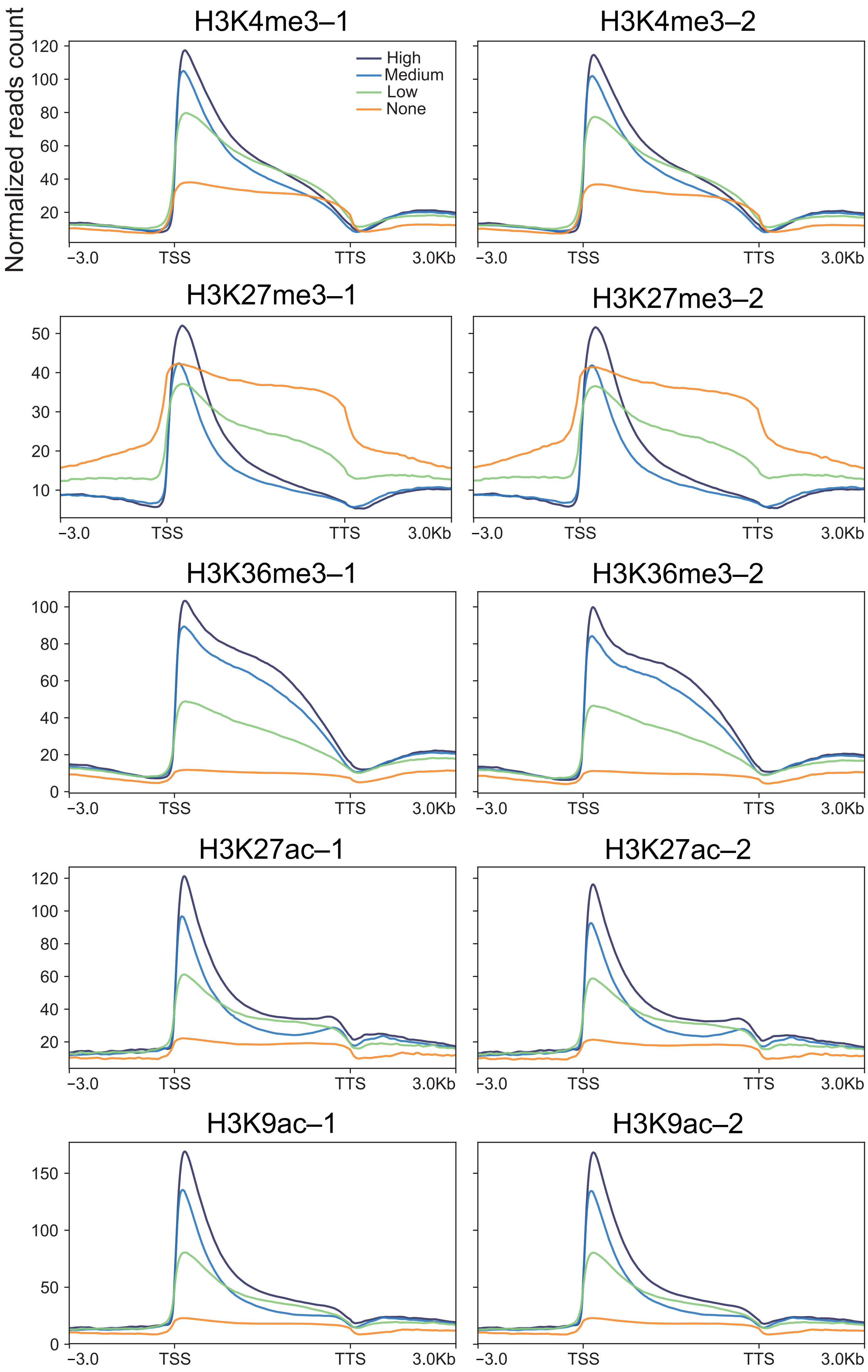

Supplementary Figure 2. Distribution of histone modifications surrounding genes. Genes were grouped based on expression levels. Expressed genes with FPKM values greater than 0.1 were equally divided into 3 groups according to expression levels (high, medium and low).

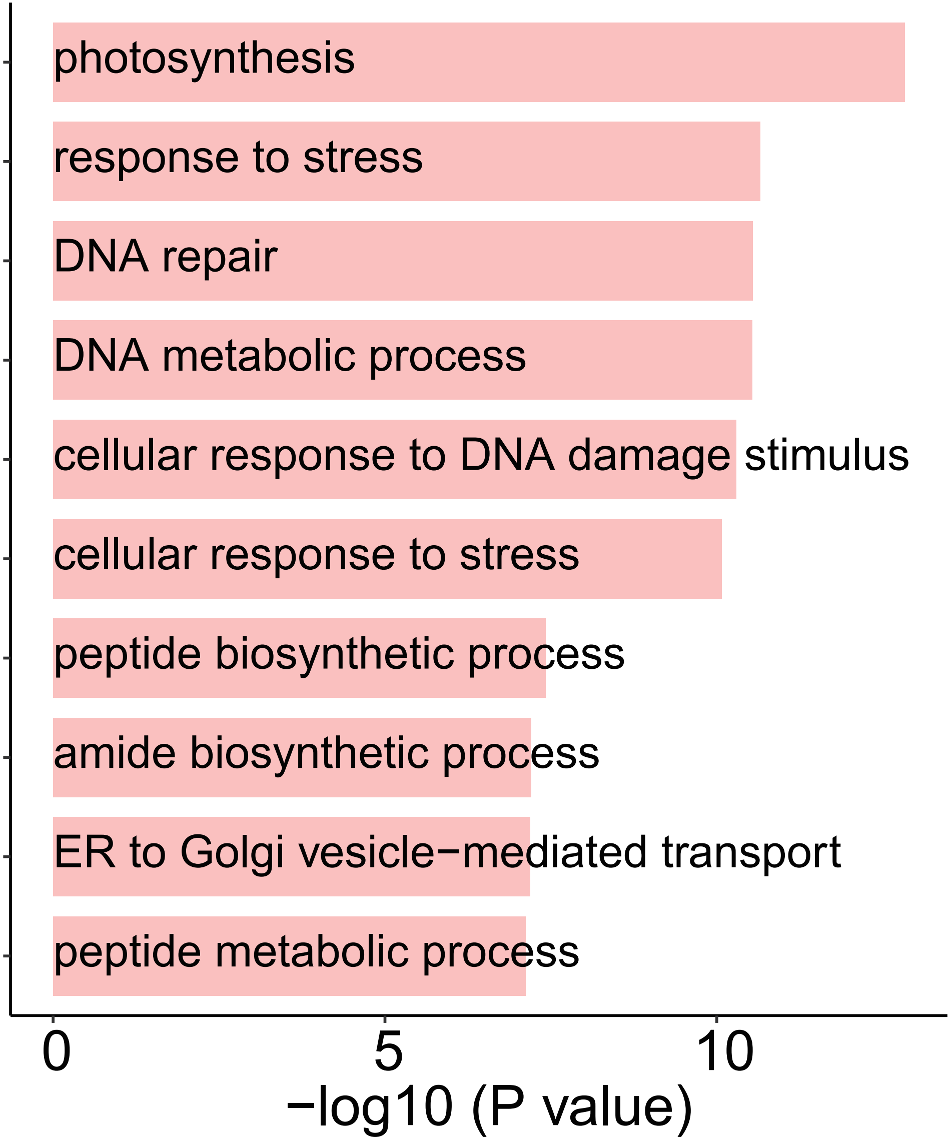

Supplementary Figure 3. GO analysis of new genes.
The top 10 enriched GO biological processes are indicated.

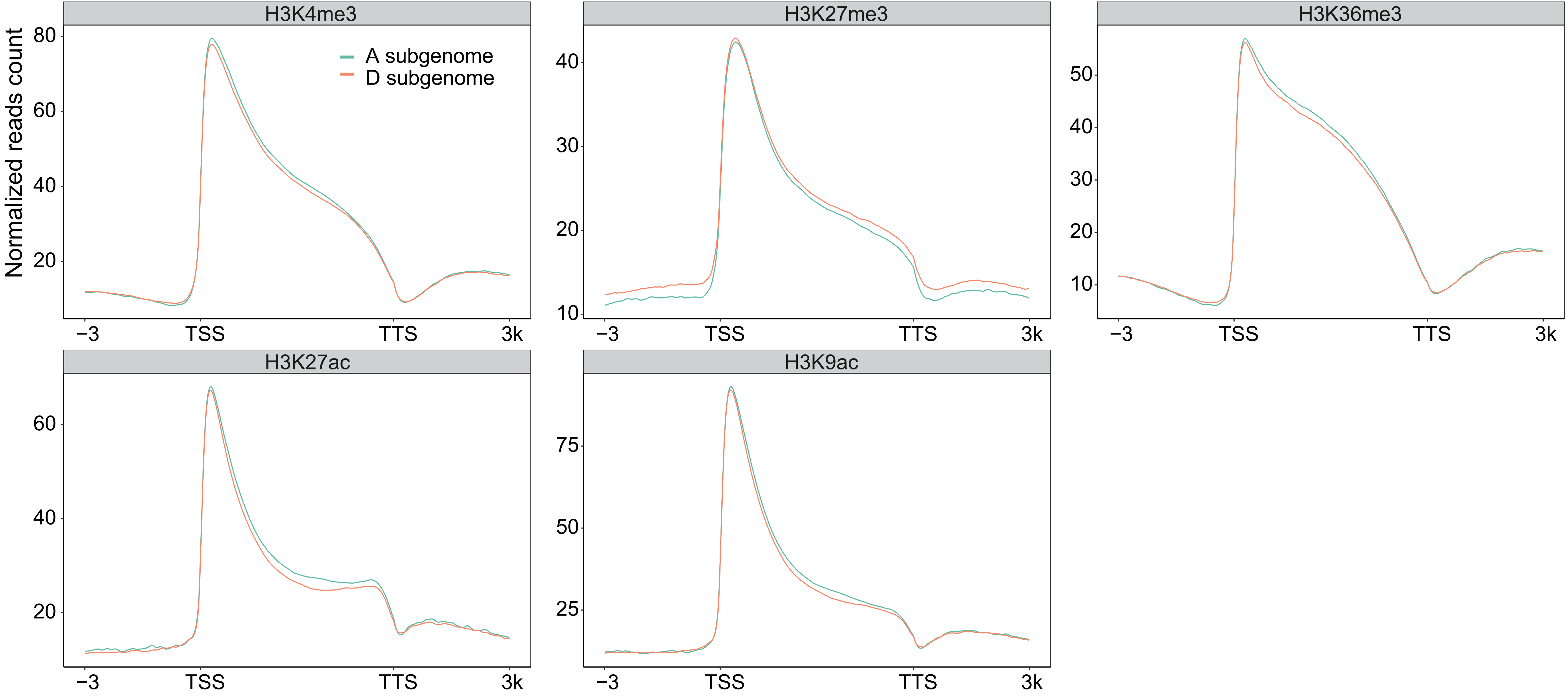

Supplementary Figure 4. Histone modifications levels of the A and D subgenomes in the genic regions.

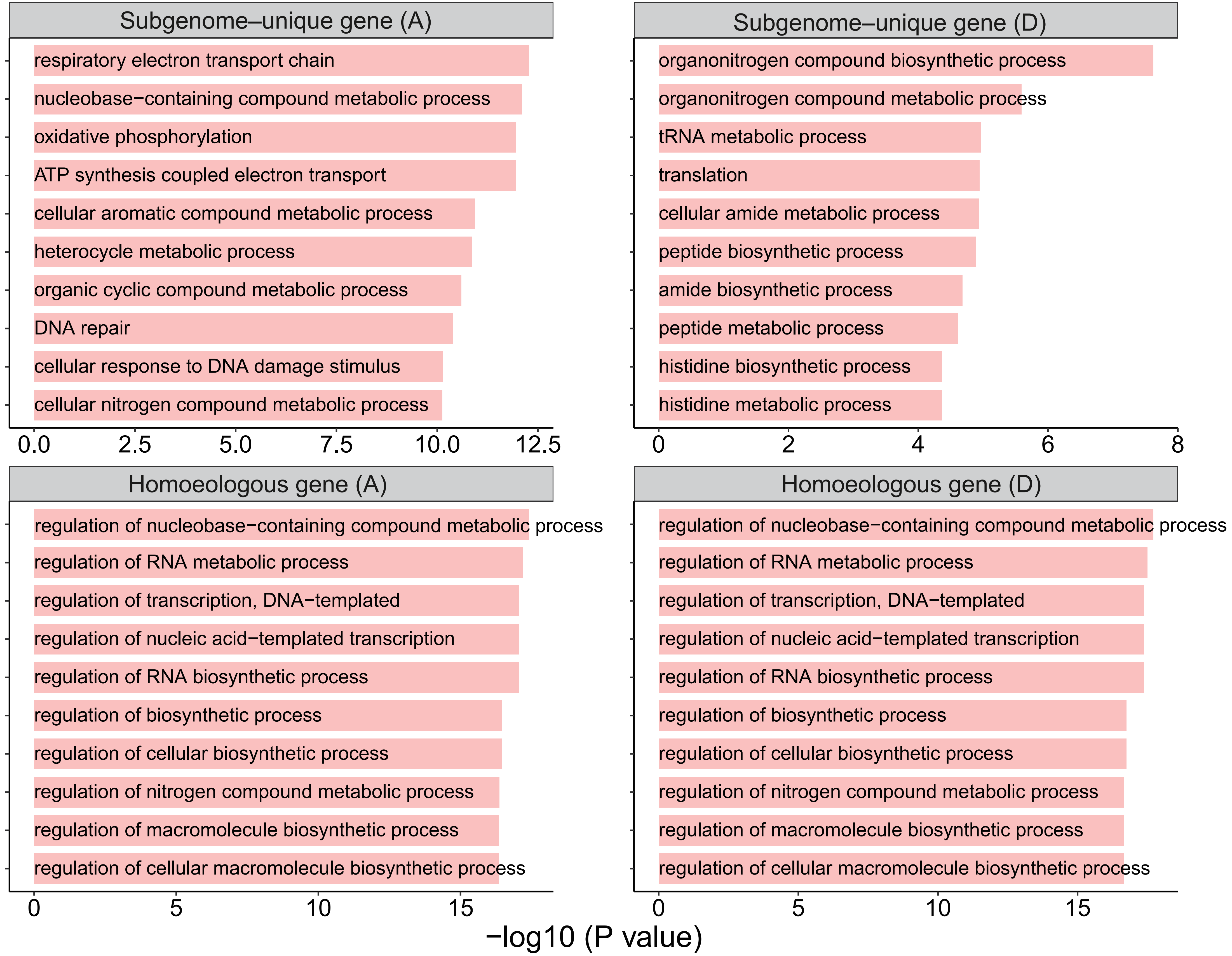

Supplementary Figure 5. GO analysis of homoeologous gene pairs and subgenome-unique genes between the A and D subgenomes.

The top 10 enriched GO biological processes are indicated.

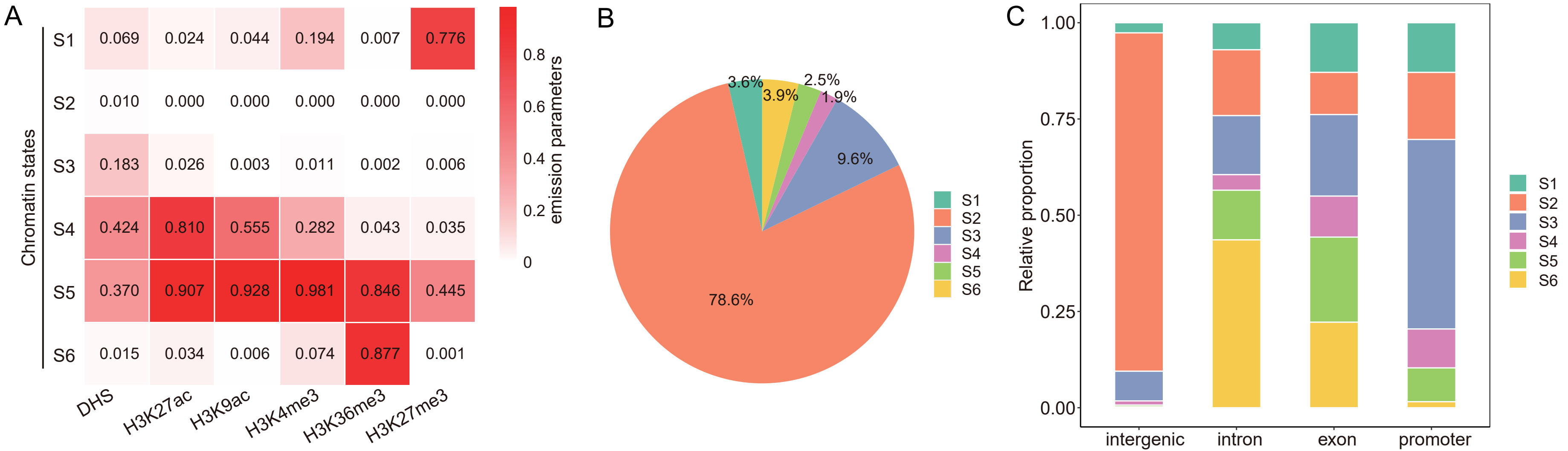

Supplementary Figure 6. Chromatin state definitions and compositions in Gh.

(A) The heatmap presents the emission parameters for each chromatin state (S1–S6).

(B) The fraction of the genome covered by each state.

(C) Relative proportion of different chromatin state across each genomic element.

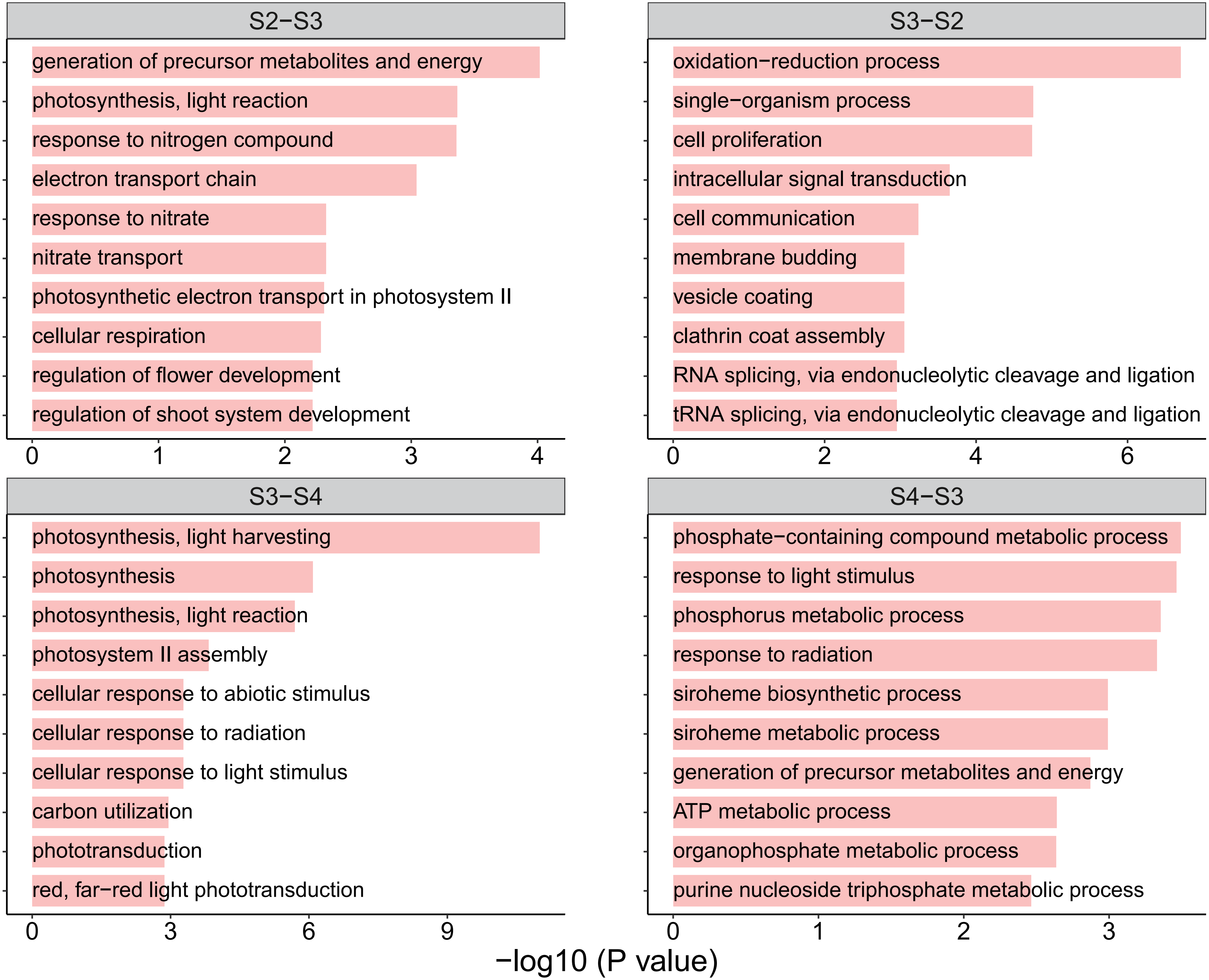

Supplementary Figure 7. GO analysis of CS transition genes.

The top 10 enriched GO biological processes are indicated.
